# Incidental Findings of Brain Magnetic Resonance Imaging Study in a Pediatric Cohort in Japan and Recommendation for a Model Management Protocol

**DOI:** 10.2188/jea.JE20090196

**Published:** 2010-03-05

**Authors:** Ayumi Seki, Hitoshi Uchiyama, Tamami Fukushi, Osamu Sakura, Koeda Tatsuya, Japan Children’s Study Group

**Affiliations:** 1 Research Institute of Science and Technology for Society, Japan Science and Technology Agency, Tokyo, Japan; 2 Department of Regional Education, Faculty of Regional Sciences, Tottori University, Tottori, Japan; 3 Department of Clinical Research, Tottori Medical Center, National Hospital Organization, Tottori, Japan; 4 Interfaculty Initiative in Information Studies, The University of Tokyo, Tokyo, Japan

**Keywords:** Brain imaging, MRI, incidental findings, research ethics, neuroethics

## Abstract

**Background:**

The increasing use of magnetic resonance imaging (MRI) in brain researches has led to growing concern over incidental findings (IFs). To establish a practical management protocol for IFs, it is useful to know the actual prevalence and problems of IF management. In the present study, we report the prevalence proportion and some handling problems of IFs in healthy Japanese children, and suggest a management protocol from ethical and practical standpoints.

**Methods:**

Between 2006 and 2008, 120 healthy children aged 5–8 years participated in a structural MRI study conducted in a pediatric cohort in Japan. All MRI images were reviewed by a pediatric neurologist, and detected IFs were classified into 4 categories.

**Results:**

IFs of all categories were detected in 40 of the 110 participants (36.4%) for whom T2-weighted or 3D-T1-weighted images were available. Findings of sinusitis and/or otitis media were most frequent (26.4%). Excluding these findings, the prevalence of IFs was still 10.9% (12 findings): 9 findings were categorized as “no referral” (8.2%), 2 as “routine referral” (1.8%), 1 as “urgent referral” (0.9%), and 0 as “immediate referral” (0.0%). In “routine referral” category, only one participant was referred for further examinations.

**Conclusions:**

Although the prevalence of IFs was high, the proportion of those requiring further examination was low. This result revealed a fairly high false-positive rate and suggested that evaluating equivocal findings was the most difficult part of IF management. A management protocol needs to include a process to properly assess the clinical importance of findings.

## INTRODUCTION

Magnetic resonance imaging (MRI) has been considered to be a low-invasive tool to assess brain structure and function. Therefore, growing numbers of researchers use MRI as a tool to study brain function and morphology in healthy subjects. Brain MRI has been used even in studies involving children. The increasing trend toward the use of MRI has led to growing concern over unexpected findings of individual brain pathology that are accidentally discovered, ie, incidental findings (IFs). In the U.S. in 2005, the National Institutes of Health (NIH), National Institute of Neurological Disorders and Stroke (NINDS), National Institute on Drug Abuse (NIDA), National Institute of Biomedical Imaging and BioEngineering (NIBIB), National Institute of Mental Health (NIMH), and National Institute on Aging (NIA) and Stanford University held a workshop with regard to this issue.^1^^,^^2^ In Japan, the Japan Neuroscience Society recently revised its Guidelines Regarding Ethical Issues of “Non-invasive Studies of Human Brain Function” to include the necessity of proper IF handling.^3^

Recently Wolf and colleagues published a comprehensive review and recommendation for managing IFs in genetic, genomic, and imaging studies.^4^ They observed that researchers have an obligation to set up a process for identifying, assessing, and communicating IFs to study participants. Although researchers are not clinicians and research scans do not aim to find pathological findings, researchers have an obligation to inform participants regarding any risks and benefits in the consent process and to minimize risks. The obligation to identify, assess, and communicate IFs is also a consequence of “researchers duties to respect the autonomy and interests of research participants” or “a reciprocity-based justification for obligations of beneficence.” Although “no action taken to IFs” is an acceptable option for researches in which images obtained are not sufficient resolution or quality to detect IFs, their policy must be approved by the IRB or ethical review committee and clearly explained to the participant in the consent process.^5^ A previous study suggested that even after participants understood that the purpose of the MRI scan was not for clinical examination, most of them still expected that findings would be detected if they existed.^6^ From an ethical viewpoint, consent for nondisclosure of IFs is not an excuse for overlooking apparent and significant findings. Researchers who use MRI as a research tool should realize the importance of IF management.

In order to establish a practical management protocol for IFs in brain MRI research, it is useful to know the actual prevalence and problems of IF management. The prevalence of IFs in brain MRI research have been reported in healthy adults,^7^ in senior adults in a population-based cohort study,^8^ in participants in a cohort study that included children,^9^ in healthy children in a cohort study,^10^ in young adult males on military flying duty,^11^ and in children in pediatric neurology clinics.^12^ The prevalence of IFs are remarkably varied among these studies, ranging from 18 to 47%.^7^^,^^9^ One of the most important factors related to this difference is the age of the population involved. Illes et al reported that the prevalence of IFs was higher in the older cohort (aged ≧60 years) than in the younger cohort (aged <60 years) although the proportion of urgent referral was higher in the younger group.^7^ Vernooij et al reported the age-specific distribution of the most frequent IFs: the prevalence of asymptomatic brain infarct and meningiomas increased with age, whereas aneurysms showed no age-related difference in prevalence.^8^ In addition to age, other differences in the backgrounds of recruited participants influenced the prevalence of IFs. In a study conducted at a pediatric neurology clinic, Gupta and Belay reported that the prevalence of IFs in patients (25.7%) was higher but that the proportion of findings that required a referral (0.3%) was much lower than in other studies.^12^ The data were collected from children with conditions for which morphological abnormalities were not usually expected, such as migraine. As is easily interpreted from these studies, the age of participants and the background for recruitment significantly affects the prevalence of IFs. However, we assume that other pragmatic factors also affect the prevalence of IFs, and it is useful to clarify these factors to establish a more effective management protocol.

Along with a pediatric cohort in Japan (Japan Children’s Study),^13^ we conducted more than 100 brain MRI scans in young children aged 5–8 years over a period of 3 years. There have been no previous report that included such a substantial number of healthy young children from a specific age group. The prevalence of IFs and characteristics of IFs in healthy young children may differ from those in adult subjects or older children. In the present study, we report the prevalence and characteristics of IFs found in healthy Japanese children aged 5–8 years and report some clinical problems we faced during the handling of IFs. We also discuss pragmatic factors that caused the difference in the prevalence of IFs, and based on those discussions, we suggest a management protocol that we developed from ethical and practical standpoints.

## METHODS

### Participants

The present study is based on brain structural MRI images obtained from participants in a cohort study of neurologically healthy children in Japan (Japan Children Study^13^). Between 2006 and 2008, 395 healthy Japanese children aged 5–8 years participated in this study. Among them, 120 children (67 boys and 53 girls) enrolled in the structural brain MRI study. The brain MRI study aimed to analyze brain volume change along with cognitive and behavioral development in healthy children.

For participation in the brain MRI study, a written proxy consent was obtained from a guardian. In the consent form, we confirmed with each guardian whether they wanted to be informed of findings that might affect the participant’s health and might need to be treated. In addition to the formal consent from a guardian, we confirmed assent for participation in the study with each child at the time of scanning.

### MRI acquisition protocol

Brain MRI scanning was performed on a Symphony 1.5T MRI scanner (SIEMENS) in the Tottori Medical Center, National Hospital Organization.

For each participant, gradient-echo T2-weighted (T2W) images and three-dimensional T1-weighted (3D-T1W) images (MPRAGE) were acquired without sedation. The acquisition times were 1 minute 40 seconds for T2W and 6 minutes 6 seconds for 3D-T1W. Some participants did not provide adequate MRI images for assessment because they could not complete the acquisition or their MRI images contained motion artifacts. Thus, only the participants for whom either T2W or 3D-T1W images were obtained were included in this study. As a consequence, 110 participants (59 boys and 41 girls) were included in the analysis.

The research protocol of the present MRI study was approved by the ethical committee of Tottori Medical Center, National Hospital Organization.

### Assessment and classification of incidental findings

All MRI images were reviewed by a pediatric neurologist on MRI scan duty on the day of acquisition. In addition, all images were retrospectively reviewed by the pediatric neurologist for this study.

IFs were classified into 4 categories as in previous reports^7^^,^^9^^,^^10^: “No referral,” “routine referral,” “urgent referral,” and “immediate referral.” No referral included common, normal findings in an asymptomatic subject, such as normal variants or minimal paranasal sinus disease. Routine referral findings required referrals for clinical assessment. This category included nonspecific white matter lesions or small cysts in extraparenchymal areas. Some previous studies^7^^,^^10^ have classified acute sinusitis into this category, but we classified all sinusitis into the no referral category because the severity of sinusitis is very difficult to assess by brain MRI without clinical information. Urgent referral findings required urgent referrals within 1 week and included nonacute intraparenchymal or extra-axial lesions. In a previous report on a pediatric population,^10^ congenital lesions or slowly progressive lesions, such as tonsillar ectopia, hypoplasia of the pons, and arachnoid cysts, were classified into this category. Immediate referral findings, such as acute processes with significant mass effects, needed to be referred immediately. Except for those cases classified as “no referral,” we reviewed how each case of IF was handled.

### Recommending a management protocol for IFs

Based on the results of the present study and previous discussions in the literature, we established a model management protocol for IFs in brain MRI studies, which will be outlined in the result (Figure 3).

## RESULTS

### The prevalence of IFs

Findings of all categories were detected in 40 of the 110 participants (36.4%) for whom either T2W or 3D-T1 images were available (Table 1). Sinusitis and/or otitis media were the most frequent findings and were detected in 29 participants (26.4%). Excluding sinusitis and/or otitis media, the prevalence of IFs was 10.9%.

**Table 1.** Incidental findings

	Total	No referral	Routine referral	Urgent referral	Immediate referral
Total	40 (36.4%)	37 (33.6%)	2 (1.8%)	1 (0.9%)	0

Extracranial	31 (28.2%)	29 (26.4%) ​ Sinusitis (28) ​ Otitis media (2)^a^	2 (1.8%) ​ Cystic lesion in the ​ sphenoidal sinus (1) ​ Polyp in maxillary sinus (1)	0	0

Intracranial	10 (9.1%)	9 (8.2%) ​ Enlarged cavum septi pellucidi ​ and cavum Vergae (6) ​ Pineal cyst (2)^a^ ​ Enlarged perivascular space (1)	0	1 (0.9%) ​ Cervical syringomyellia (1)	0

^a^One participant had both findings.

**Figure 1.**
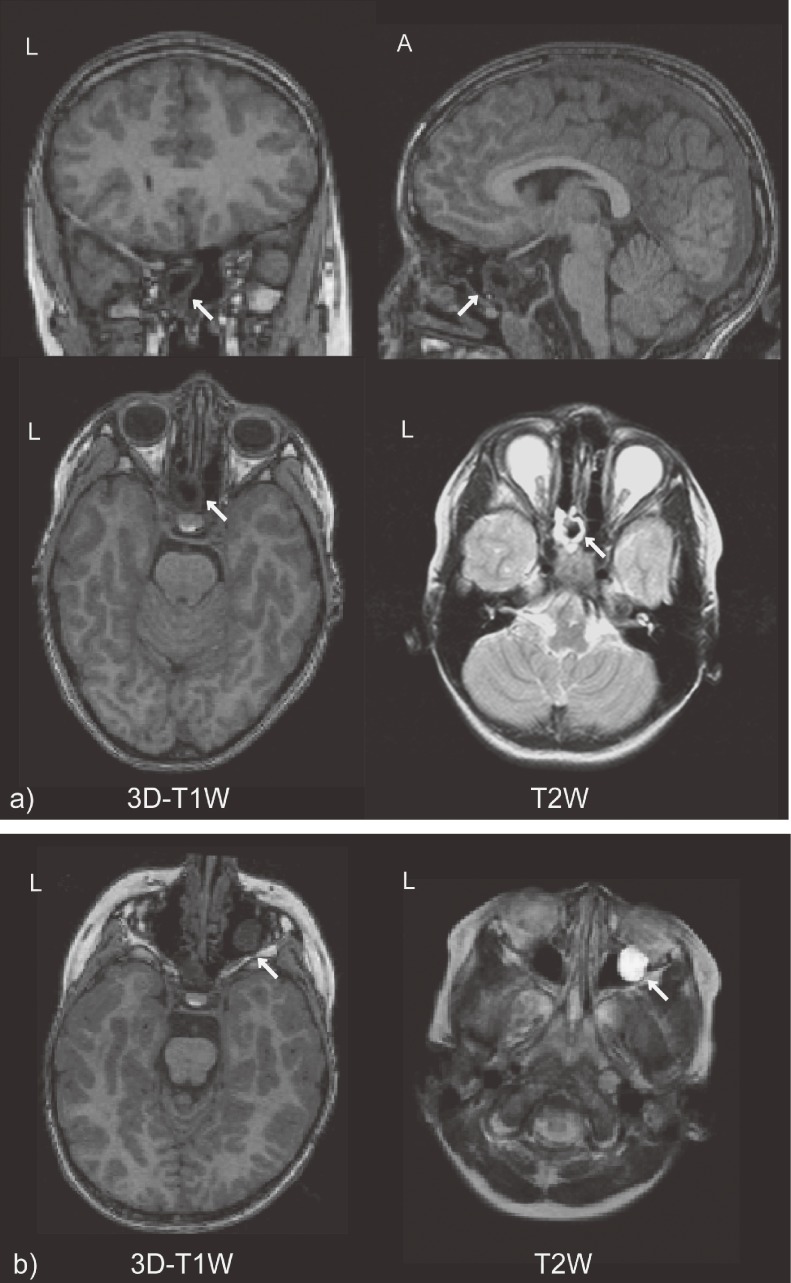
Incidental findings classified into routine referral category. a) Cystic lesion in the sphenoidal sinus, b) polyp in maxillary sinus. (A: anterior, L: left, 3D-T1W: D-T1 weighted image, T2W: T2 weighted image)

**Figure 2.**
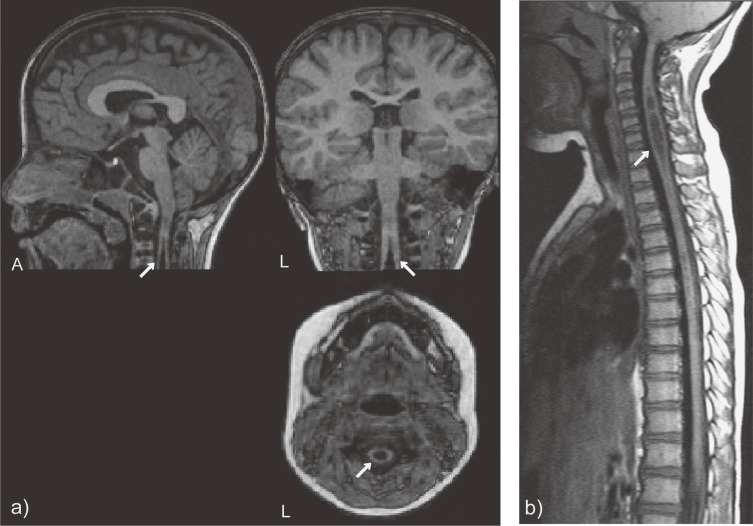
A incidental finding classified into routine referral category: Cervical syringomyelia. a) 3D-T1 weighted images obtained in our research, b) Spinal MRI acquired as further examination after referral. Arrows indicate the lesions. (A: anterior, L: left)

Nine participants (8.2%) were classified into the “no referral” category with enlarged cavum septi pellucid and cavum vergae (in 6 participants), pineal cyst (in 2 participants), and enlarged perivascular spaces (in 1 participant). Two participants (1.8%) were classified into the routine referral category with a cystic lesion in the sphenoidal sinus (Figure 1a ) and a polyp in the maxillary sinus (Figure 1b). One participant (0.9%) with cervical syringomyelia was classified into the urgent referral category (Figure 2a ). No participants were classified into the immediate referral category.

### The management process for each IF

In the two findings classified into routine referral category, a cystic lesion in the sphenoidal sinus (Figure 1a) needed the 3D-T1W images to be checked from multiple directions to clarify its location and relation to the surrounding central nervous system. We confirmed that the cyst was fully isolated in the sphenoidal sinus, and therefore, we did not refer it to another clinic for further examination. However, we informed his guardian of the existence of the finding and explained our interpretation: The finding was unusual but did not require treatment. No related symptom has occurred in this participant in the 2 years since the MRI scan. On the other hand, we advised the other participant with IF in the routine referral category—a polyp in the maxillary sinus (Figure 1b)—to see an otorhinolaryngologist because we thought it might need treatment. Consequently, it was diagnosed as an asymptomatic maxillary polyp and no further follow-up was required.

In the case of the urgent referral finding—syringomyelia—we informed the child’s parents of the finding on the day of the MRI scan and recommended that they visit a neuropediatrician for further examinations. The brain and spine MRI conducted under sedation revealed that the lesion was large (Figure 2b), and the participant was referred further to a neurosurgeon. He is under regular observation by the neurosurgeon, although no clinical symptoms have emerged.

### A recommended management protocol for IFs

The present study revealed two important facts associated with IFs in young children. First, the prevalence of IFs excluding sinusitis and/or otitis media was somewhat high (10.9%) compared to previous reports, but the proportion of those requiring further examination was rather low (0.9%). Second, the two participants with findings classified into the routine referral category were managed differently: Although IFs were disclosed in both cases, one was referred and the other was not. These facts indicated that the false-positive rate of IFs was fairly high and that evaluating equivocal findings was the most difficult part of IF management.

**Figure 3.**
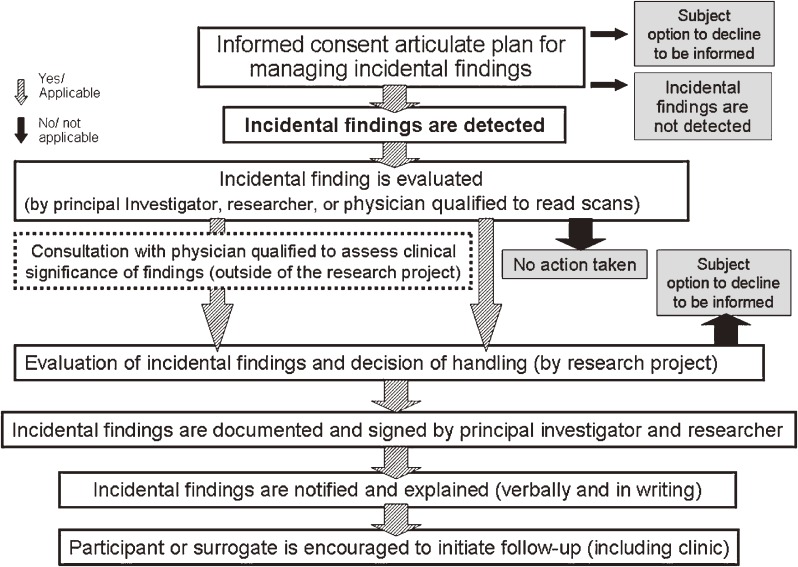
The recommended IF management protocol used by the Japan Children’s Study Group.

Based on these results, the Japan Children’s Study Group developed a management protocol for IFs, as shown in Figure 3. For evaluation of IFs, producing a management manual for each finding is not likely to be practical because of the numbers and variety of findings. Our protocol includes a procedure whereby investigators can consult with specialists outside of the research team before they decide whether to disclose findings to participants (dotted-line box). Showing data obtained in the research to personnel who are outside of the research team may be problematic from the viewpoint of privacy protection. Therefore, we explained the possibility that we might show MRI images to other experts for consultation to participants during the consent process and treated data anonymously during the consultation. After taking the opinions of the outside experts into consideration, the decision of how to handle each IF is made by plural members of the research team; however, the principal investigator of the research team takes responsibility for the decision. Disclosing IFs to guardians (and participants) is conducted orally and is followed up with a written form confirming the statement on the consent form. The researcher who is already in contact with the participant is charged with making the disclosure and advises them as to the next step.

## DISCUSSION

### The difference in the prevalence of IFs

The prevalence of IFs in the present study of healthy children aged 5–8 years was remarkably high (36.4%; 40 out of 110 participants); however, the most frequent findings were sinusitis and/or otitis media, which are common conditions in young children. MRI can detect subtle changes caused by this disease, and the predominance of sinusitis and otitis media is undoubtedly related to the age of participants in the study. Excluding sinusitis and otitis media, the prevalence of IFs in our study was 10.9%, which was still high compared to previous reports, although only one case (0.9%) required further medical follow-up.

The prevalence of IFs varies widely among reports. One of the most important factors is the character of the study population, such as the age range of participants and the location where participants are recruited. Another important factor is the difference in scan protocols and the reviewers’ backgrounds. In the research setting, the scan protocol may not suitable for detecting and evaluating IFs, and reviewers have less experience in judging the clinical significance of findings. In addition, the difference in the criteria for IFs is not negligible. Gupta and Belay, who reported the prevalence of IFs at a pediatric neurology clinic, proposed a different classification of IFs based on the requirements for a change in management.^12^ A clinician’s viewpoint is focused on whether the detected findings require a change in management, while the viewpoint of a researcher is focused on whether they require further expert review or additional examinations to judge clinical significance.

Considering differences among such possible factors in each study, the diversity of prevalence among studies is understandable. It is important for researchers to know both the possibility and limitations of detecting IFs in each study protocol and to explain them to participants in the consent process. In our study, a clinician reviewed all images obtained for research purposes. The participants may have more expectation that any abnormal findings should be detected if the reviewers are clinicians. Most previous reports agreed that additional scans for clinical evaluation of IFs should not be obtained within the research protocol.^2^ The limitations for detecting abnormal findings resulting from the scanning protocol should be mentioned explicitly during the consent process.

### Disclosure of IFs to participants

Overlooking significant IFs is the most critical scenario. On the other hand, considering the high rate of pseudopositive findings in brain MRI, disclosing all findings to participants is another ethical problem because disclosing IFs may cause anxiety and a financial burden if further clinical examinations are necessary.

Wolf et al insist that IF should be disclosed to the research participants only when the benefit of knowing the IF exceeds that of not knowing.^4^ They recommend a classification of IFs based on the benefit of disclosing the IFs to the research participants: “strong net benefit,” “possible net benefit,” and “unlikely net benefit”. “Strong net benefit” includes conditions likely to be grave or life-threatening that can be avoided or ameliorated; to disclose IFs in this category is markedly more of a benefit than a burden. Researchers have an obligation to disclose IFs in this category. “Possible net benefit” includes nonfatal conditions that are likely to be grave but cannot be avoided or ameliorated. IFs in this category may be disclosed to research participants depending on participants’ informational needs and preferences. The category “unlikely net benefit” includes conditions that are not likely to be of serious health importance or whose health importance cannot be ascertained. This information should not be disclosed because it is more of a burden than a benefit for participants. Wolf et al also recommended that the level of information participants want to know should be confirmed in advance.^4^ A right not to know should be respected, but they recommend that it should be reconfirmed with a participant when the IF is life-threatening or grave and treatable.

In our cases, the findings classified into the urgent referral category required further examinations and follow-up, and therefore, it corresponds to “strong net benefit”. In 2 cases classified into the routine referral category, we referred 1 participant to another expert but did not refer the other, only informing that participant of the finding. Consequently, both participants did not require further treatment or follow-up. For the former case, disclosure of the IF may have levied some psychological and financial burden on the participant to see a physician who was not consequently needed. For the latter case, the participant had no financial burden, but disclosing the IF without any follow-up may have caused anxiety. Adapting the Wolf classification, both IFs corresponded to “unlikely net benefit” in the end, but at the time of review, either of them could have been a treatable finding, and in accordance with this meaning, both could have been categorized as “strong net benefit”. Their classification of IFs gives the guidelines for disclosing IFs to participants but does not reduce the difficulty in judgment. For researchers who conduct brain MRI studies, a more practical approach for assessment is required.

### A recommended IF management protocol

The most noteworthy aspect of our recommended management protocol (Figure 3) is that it defines the evaluation process including consultation with specialists outside of the research team (a dotted-line box). In the pathway for handling IFs recommended by Wolf et al, the consultation with experts is included.^4^ The consultation is utilized to determine the categories of each IF based on the benefit or burden of disclosing it. However, it is highly difficult even for experts to decide the benefit or burden of disclosing an IF due to limitations of the scan and lack of clinical information. Our consultation with experts aims to evaluate the need of referrals for further examinations. Experts may suggest a further referral because of insufficiency of imaging quality or information. If the IF turns out to not require further examination or treatment after a participant visits a physician, it only imposes a psychological and financial burden on the participant. Researchers should inform participants about this issue and obtain consent based on that understanding.

We suggest that the decision regarding management of each IF should be made by multiple members of the research team after considering the advice of experts and that the principal investigator of the research team should bear final responsibility for the decision. It is clear that the responsibility for managing IFs lies with the primary investigator and not on those conducting the research scans or on reviewers who are not core members of the research team.^4^

As we reported in this study, the prevalence of IFs in brain imaging studies is not negligible. Compounding the risk to participants and researchers of overlooking significant IFs, unexpectedly encountering significant IFs will be an additional psychological burden for researchers who do not have clinical experience. Being aware of IFs and preparing for them is the responsibility of the primary investigators of brain imaging studies, and this extends to both research participants and members of the research team.

### Overall discussion

In the present study, we reported the prevalence of IFs in healthy children aged 5–8 years, reviewed the management process for each case with IFs, and on the basis of these results and on previous discussions in the literatures, we established a model management protocol for IFs in brain MRI studies.

It is important for each study team to prepare a management protocol for IFs, but the involvement of IRBs and the guidance of governments is also integral.^4^ The IRB has a crucial role in assuring that each research protocol sets up an appropriate plan for managing IFs.^4^ The research community in Japan, however, is presently less aware of the importance of appropriate management of IFs.^14^ Currently in Japan, the Guidelines Regarding Ethical Issues of “Non-invasive Studies of Human Brain Function” of the Japan Neuroscience Society is the only standard for managing IFs in MRI studies.^3^ The newly revised guidelines cover important issues related to IFs in MRI research as we have discussed in the present report, and we highly recommend that researchers who conduct brain imaging research follow these guidelines.

In human research, consideration of the research protocol from an ethical point of view is important not only to protect the rights of participants or to defend researchers from risks, it is also important for developing mutual confidence between researchers and participants and for consequentially increasing the public’s trust of human research.
